# DEMENTIA DIAGNOSES PREDICT CAREGIVER EDUCATION DISCREPANCIES IN HOSPITAL SETTING

**DOI:** 10.1093/geroni/igad104.0462

**Published:** 2023-12-21

**Authors:** Austin Medlin, Catherine Still, Nicole Werner, Beth Fields

**Affiliations:** Indiana University, Bloomington, Indiana, United States; University of Wisconsin-Madison, Madison, Wisconsin, United States; Indiana University School of Public Health-Bloomington, Bloomington, Indiana, United States; University of Wisconsin-Madison, Madison, Wisconsin, United States

## Abstract

Many family caregivers report feeling unprepared to provide care for persons living with dementia (PLWD) after their hospital stay. Consequently, PLWD may experience poorer health outcomes and higher service utilization rates. Health systems can provide education to patients and their family caregivers, increasing preparedness, but these practices are less understood. This retrospective data analysis of electronic health records examines the role of diagnostic billing codes in predicting amount of education received by patients and their family caregivers. Our dataset includes 419 unique encounters of hospitalized older adult patients with a documented dementia diagnosis and a listed family caregiver between 1/1/2019 and 8/1/2022. Dementia-related ICD-10 billing codes determined unique patient diagnoses. Encounters were given designations of receiving more vs. less education if the amount of education received was above or below the sample median. Logistic regressions then determined the relationship between diagnostic billing codes and education received while controlling for demographic and contextual characteristics. We found that encounters with diagnostic codes “other degenerative disease of nervous system” and “Alzheimer’s disease” were 2.8 and 3.3 times as likely to receive more education, respectively (95% confidence interval = [1.051, 7.347], p = .0394; [1.175, 9.461], p = .0236). Conversely, encounters with the code “other frontotemporal dementia” were 3.3 times as likely to receive less education ([1.150, 9.684], p = .0266). Findings demonstrate certain dementia-related diagnoses predict differences in how likely caregivers are to receive education. Health systems should evaluate for these differences and incorporate new methods to increase caregiving preparedness.

